# House spider genome uncovers evolutionary shifts in the diversity and expression of black widow venom proteins associated with extreme toxicity

**DOI:** 10.1186/s12864-017-3551-7

**Published:** 2017-02-16

**Authors:** Kerry L. Gendreau, Robert A. Haney, Evelyn E. Schwager, Torsten Wierschin, Mario Stanke, Stephen Richards, Jessica E. Garb

**Affiliations:** 1 Department of Biological Sciences, University of Massachusetts Lowell, Lowell, MA 01854 USA; 2 Department of Biological Sciences, Virginia Tech, Biocomplexity Institute, Blacksburg, VA 24061 USA; 3 Institut für Mathematik und Informatik, Ernst-Moritz-Arndt Universität Greifswald, Walther-Rathenau-Straße 47, 17487 Greifswald, Germany; 4 Human Genome Sequencing Center, Department of Human and Molecular Genetics, Baylor College of Medicine, Houston, TX 77030 USA

**Keywords:** *Latrodectus*, Venom toxins, Gene family evolution, Genomics, RNA-Seq

## Abstract

**Background:**

Black widow spiders are infamous for their neurotoxic venom, which can cause extreme and long-lasting pain. This unusual venom is dominated by latrotoxins and latrodectins, two protein families virtually unknown outside of the black widow genus *Latrodectus,* that are difficult to study given the paucity of spider genomes. Using tissue-, sex- and stage-specific expression data, we analyzed the recently sequenced genome of the house spider (*Parasteatoda tepidariorum*), a close relative of black widows, to investigate latrotoxin and latrodectin diversity, expression and evolution.

**Results:**

We discovered at least 47 latrotoxin genes in the house spider genome, many of which are tandem-arrayed. Latrotoxins vary extensively in predicted structural domains and expression, implying their significant functional diversification. Phylogenetic analyses show latrotoxins have substantially duplicated after the *Latrodectus/Parasteatoda* split and that they are also related to proteins found in endosymbiotic bacteria. Latrodectin genes are less numerous than latrotoxins, but analyses show their recruitment for venom function from neuropeptide hormone genes following duplication, inversion and domain truncation. While latrodectins and other peptides are highly expressed in house spider and black widow venom glands, latrotoxins account for a far smaller percentage of house spider venom gland expression.

**Conclusions:**

The house spider genome sequence provides novel insights into the evolution of venom toxins once considered unique to black widows. Our results greatly expand the size of the latrotoxin gene family, reinforce its narrow phylogenetic distribution, and provide additional evidence for the lateral transfer of latrotoxins between spiders and bacterial endosymbionts. Moreover, we strengthen the evidence for the evolution of latrodectin venom genes from the ecdysozoan Ion Transport Peptide (ITP)/Crustacean Hyperglycemic Hormone (CHH) neuropeptide superfamily. The lower expression of latrotoxins in house spiders relative to black widows, along with the absence of a vertebrate-targeting α-latrotoxin gene in the house spider genome, may account for the extreme potency of black widow venom.

**Electronic supplementary material:**

The online version of this article (doi:10.1186/s12864-017-3551-7) contains supplementary material, which is available to authorized users.

## Background

Animal venoms attract wide scientific attention because of their biomedical applications and are an excellent model for understanding the origins and diversification of ecologically important genes [1–3]. Venoms are largely composed of diverse proteins and peptides, and while proteomic and transcriptomic analyses have substantially advanced knowledge of venom composition, few studies have focused on venom at the genomic level [3]. Studies that have utilized the genomes of venomous animals, including those of the king cobra, platypus, scorpion, and velvet spider, have been particularly helpful in elucidating mechanisms of venom gene recruitment, gene family expansion, and a genetic basis for venom self-resistance [4–7]. Yet limited or no genomic data is available for some of the most medically important venomous species, obstructing insights into the evolution of especially dangerous toxins.

The development of rapid and cost-effective next generation sequencing (NGS) technologies has launched many new whole genome projects focused on biomedically or agriculturally important eukaryotes. An example of such efforts is the i5k (5000 arthropod genomes) initiative [8], which has recently sequenced the house spider (*Parasteatoda tepidariorum*) genome (NCBI Accession GCA_000365465.1). This species is an emerging developmental model that is in the same family (Theridiidae) as black widow spiders (*Latrodectus* spp.), but produces far less hazardous venom [9, 10]. Together with extensive tissue- and stage-specific transcriptomic data, the house spider genome provides an important resource to investigate the evolutionary basis for the extreme toxicity of black widow venom.

The venoms of *Latrodectus*, a genus that includes multiple spider species referred to as black widows and the Australian red-back, are notable for their potency and ability to cause extreme pain in humans lasting for days in addition to systemic effects such as muscle spasms, difficulty breathing and paralysis [11, 12]. The severe symptoms of widow spider envenomation in vertebrates are largely attributed to α-latrotoxin, a member of a unique family of large neurotoxins that form exogenous calcium channels in the neuronal pre-synaptic membranes of injected victims, causing massive neurotransmitter release [13–15]. In addition to α-latrotoxin, three other latrotoxin genes have been functionally analyzed from *Latrodectus* species: α-latroinsectotoxin and δ-latroinsectotoxin, which have toxic effects on insects but not vertebrates, and α-latrocrustotoxin, which is toxic to crustaceans [16–20]. More recently, NGS technology has been used to identify at least 20 unique latrotoxins expressed in black widow venom glands [21, 22]. While the diversity and evolution of the latrotoxin gene family remains poorly understood, Zhang et al. [23] linked their origin in spiders to lateral gene transfer between spiders and endosymbiotic bacteria.

In addition to latrotoxins, black widow venom contains a second unique protein family termed latrodectins (also known as latrotoxin associated low molecular weight proteins - LMWPs) that co-purify with latrotoxins and are highly expressed in venom glands. Latrodectins are not toxic to mice or insects on their own but evidence suggests that they contribute to latrotoxin toxicity [24–28]. Previous evolutionary analyses support the recruitment of latrodectins for expression in black widow venom from a gene in the ecdysozoan neuropeptide superfamily containing crustacean hyperglycemic hormones (CHH) and ion transport peptides (ITP) [25, 29, 30]. Genomic sequences from the house spider can generate new insight into the diversity and evolution of venom latrodectin genes in relation to the broadly expressed CHH/ITP superfamily genes.

Despite the relatively close evolutionary relationship between *Latrodectus* and *Parasteatoda*, house spider envenomation of humans is far less severe than black widow envenomation [10]. While *Parasteatoda* bites to humans may also cause neurotoxic (albeit far less severe) pain, they do not result in the debilitating systemic effects (nausea, cramps, etc.) associated with *Latrodectus* bites [10]. Protein gel analyses of *P. tepidariorum* venom also showed an electrophoretically distinct profile from black widow venom, lacking proteins in the size range of the latrotoxins [31]*.* Together, this evidence implies that evolutionary transformations of venom protein composition, involving changes in latrotoxin structure or expression, may account for the greater potency of black widow venom in comparison to house spider venom. Accordingly, we used the house spider genome and multi-tissue and stage-specific expression data to investigate the diversity, evolution and relative expression of latrotoxins and latrodectins due to their abundance and functional importance in black widow venom. Our results provide strong evidence for the evolution of venom-expressed latrodectins through tandem duplication and neofunctionalization of the non-venom CHH and ITP genes. We also substantially expand the functional diversity of the medically important latrotoxin family and provide further evidence for a potential lateral gene transfer of latrotoxins with a bacterial endosymbiont. Additionally, we show the greater expression of latrotoxins in black widow venom glands relative to house spider venom glands, which, along with the lack of a α-latrotoxin ortholog, provides a molecular explanation for the greater potency of black widow venom toward vertebrates.

## Results

### House spider genome encodes numerous diverse latrotoxin genes

**Fig. 1:**
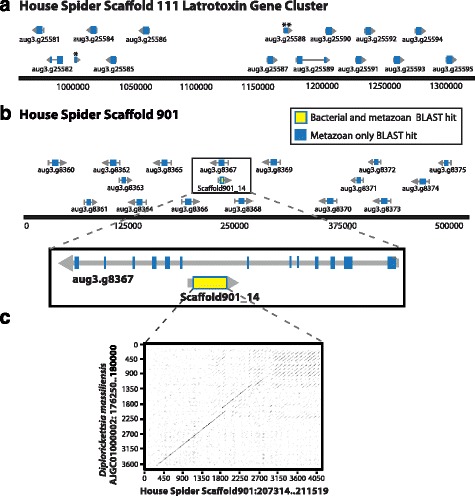
House spider genome contains clusters of latrotoxin genes and suggests their relationship with bacterial genes. **a** *Parasteatoda tepidariorum* (house spider) genomic scaffold 111 contains 14 tandem latrotoxin genes (*blue rectangles*) in a 300 kb region; * non-latrotoxin gene (putative transposable element); ** fragmented latrotoxin gene. **b** Latrotoxin gene (labeled Scaffold901_14) on opposite strand embedded within intron of gene aug3.g8367. The translation of scaffold901_14 (*yellow region*) has significant BLASTp hits to proteins from the endosymbiotic bacterium *Diplorickettsia massiliensis* (*e*-values ranging from 6 × 10^−17^ to 6 × 10^−59^); all other proteins encoded on this scaffold only have significant BLASTp hits to metazoan sequences (*blue*). **c** Dotplot showing alignment of house spider gene 901_14 that shares significant BLASTn homology (*e*-value 0.0, 70% identity) to ~1500 bp of the *D. massiliensis* genome. Please see Additional file 9 for a high resolution version of this figure

We identified at least 47 latrotoxin coding genes on 23 scaffolds of the common house spider genome (Additional file 1). We found six additional sequences with significant similarity to latrotoxins that were not included in phylogenetic analyses due to their shorter lengths in comparison to known latrotoxins. These sequences likely represent latrotoxins that have not been sequenced completely (most are interrupted by N’s or are at scaffold ends). Twenty-one of the 47 full-length latrotoxin genes are distributed in tandem along two genomic scaffolds with as many as 14 tandem latrotoxin genes spanning ~300 kb of scaffold 111 and 7 spanning ~120 kb of scaffold 1821 (Fig. 1a). A putative latrotoxin gene not recognized by Augustus, but found through our BLAST searches (labeled Scaffold901_14) and labeled as PtepTmpM012796-RA in the Baylor College of Medicine MAKER annotations (https://i5k.nal.usda.gov/annotations/85472), is on a different scaffold (901) and is embedded in the intron of another gene (aug3.g8367.t1) on the opposite strand (Fig. 1b). The protein of this surrounding gene (containing a latrotoxin gene within its intron) has a significant BLAST hit to calcium-binding protein 1 (Genbank accession KFM83056.1).

Translated house spider latrotoxin genes show substantial sequence divergence, with pairwise percent identities across sequences in our trimmed alignment ranging from 27 to 91%. The lengths of these latrotoxins range from 972 to 1404 amino acids and vary in the numbers and types of their predicted functional domains (Additional file 1). The number of predicted ankyrin repeats ranges from 6 to 19 with one exception (the intron-embedded Scaffold 901_14 latrotoxin contains 25 ankyrin repeats). Known black widow latrotoxins have a smaller range of predicted ankyrin repeats – between 11 and 20 [22]. Additionally, the average number of predicted ankyrin repeats is significantly higher in *Latrodectus* and *Steatoda* (*Latrodectus*’ sister genus) latrotoxins (15) than in house spider latrotoxins (12), with a Student’s *t*-test *p*-value of 3.0 × 10^−4^. A latrotoxin C-terminal domain (CTD) is predicted in all but four complete house spider latrotoxin sequences. Additional functional domains were predicted in house spider latrotoxin sequences, including one or more transmembrane and coiled-coil domains in both latrotoxin N- and C-termini. One latrotoxin protein– aug3.g26325 – contains two transmembrane domains as well as a t-SNARE domain spanning its N terminus.

### House spider latrotoxin shares significant nucleotide identity with arachnid bacterial endosymbiont

No protein predicted from the more distantly related velvet spider (*Stegodyphus mimosarum*) genome had significant BLAST homology to the conserved latrotoxin N-terminal or the latrotoxin CTD, however, a BLASTp search of the NCBI nr database using the first 320 amino acids (containing the N-terminal region) of house and widow spider latrotoxins resulted in four non-spider protein sequences (WP_010598965, WP_010598284, WP_010598285, and WP_010598286) with significant hits (*e*-values ranging from 6 × 10^−17^ to 7 × 10^−59^). All of these proteins are encoded by the genome of *Diplorickettsia massiliensis*, a bacterial endosymbiont of *Ixodes ricinus* ticks [32]. Of the latrotoxin queries used for this search, Scaffold901_14 (embedded in the intron of another gene, aug3.g8367.t1, described above) had the most significant BLAST hits to *D. massiliensis* sequences. Therefore, we used the Scaffold901_14 gene as a query for a BLASTn search of the *D. massiliensis* genome. This search revealed a region of the *D. massiliensis* genome with high sequence identity to a region on scaffold 901 containing a portion of the Scaffold901_14 gene (sharing 70% identity over >1500 nucleotides; Fig. 1c). Based on the initial annotation, this region in *D. massiliensis*, spanning bases 178232 to 179776 on genomic scaffold AJGC01000002.1, contains consecutive genes encoding three of the protein sequences found with BLASTp (WP_010598284, WP_010598285, and WP_010598286). Additional annotation from the Pathosystems Resource Integration Center (PATRIC) includes six predicted genes in this genomic region (fig|1156986.4.peg.1434, fig|1156986.4.peg.1435, fig|1156986.4.peg.1436, fig|1156986.4.peg.1437, fig|1156986.4.peg.1438, fig|1156986.4.peg.1439; [33]). We combined the protein translations of these six predicted bacterial genes into a single sequence and aligned them with the house and widow spider latrotoxins, along with *D. massiliensis* protein WP_010598965, the additional sequence with a significant BLASTp hit to latrotoxins. Scaffold901_14 was the only gene on scaffold 901 whose product had significant BLAST homology to a bacterial sequence (Fig. 1b; Additional file 2).

### Lineage-specific expansion of house spider and black widow latrotoxins

**Fig. 2:**
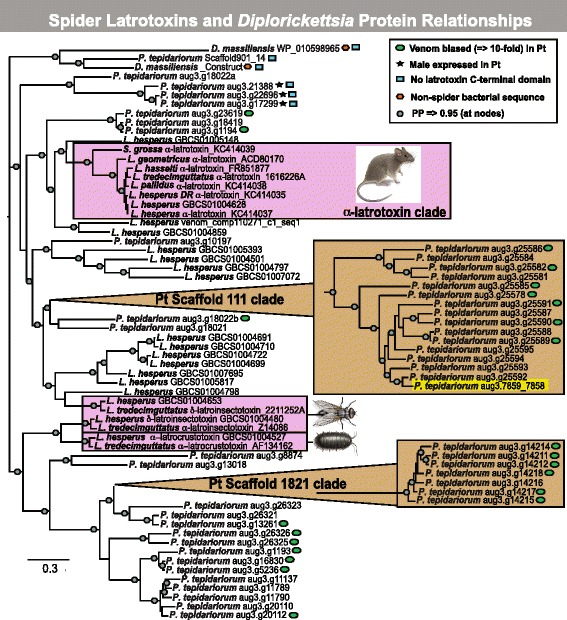
Latrotoxins experienced substantial gene duplication and diversification in cob-web weaving spiders (Theridiidae). A Bayesian 50% majority rule consensus tree rooted at the midpoint showing relationships of black widow (*Latrodectus*) and common house spider (*Parasteatoda tepidariorum*) latrotoxins, along with *Diplorickettsia massiliensis* bacterial proteins (indicated by an *orange polygon*). Nodes with posterior probability = > 0.95 indicated by *gray dots*; *pink boxes* surround functionally characterized black widow venom latrotoxins, with adjacent images representing toxicity targets (mouse for vertebrate toxin α-latrotoxin; fly for insect toxins; isopod for crustacean toxin; images from Emily Damstra). Pt = *Parasteatoda tepidariorum*; Pt (genomic) scaffold 111 clade includes aug3.g7859_7858 (in *yellow*) on a separate scaffold, sequences labeled male expressed (*black star*) if expression in whole males was higher than in all other tissues (venom, silk, ovaries, whole females, and two developmental stages, in TPM). Pt sequences with venom-gland biased expression indicated by *green ovals*. Non *P. tepidariorum* sequences labeled by their GenBank accession numbers. Please see Additional file 9 for a high resolution version of this figure

Bayesian phylogenetic analysis of latrotoxins largely grouped sequences into species-specific clades (Fig. 2). With few exceptions, large clades of house spider latrotoxins do not include sequences from *Latrodectus* species, indicating that the latrotoxin gene family has undergone substantial lineage-specific duplication in descendants of the most recent common ancestor of *Parasteatoda* and *Latrodectus* (estimated to have existed 90 mya [34]). For example, no ortholog of the vertebrate neurotoxin α-latrotoxin was found in the house spider genome. α-Latrotoxin from *Latrodectus* and *Steatoda* are instead more closely related to three paralogs from *L. hesperus* (Fig. 2, PP = 1.0). Generally, house spider latrotoxins nearest one another on genomic scaffolds are more closely related to each other than to latrotoxins from other scaffolds, consistent with many local, recent gene duplication events. For example, 15 latrotoxins from scaffold 111 are in a clade along with only one sequence (aug3.g7859_7858) from scaffold 846 (PP = 1.0; Fig. 1a, Fig. 2). Gene aug3.g7859_7858 (a concatenation of two incorrectly annotated genes) is most closely related to aug3.g25592 on scaffold 111 and they share 74% protein identity over their entire lengths, suggesting past duplication events occurring across more distant genomic regions. The house spider latrotoxin Scaffold901_14 forms a well-supported clade (PP = 1.0) with the two included *D. massiliensis* sequences (protein WP_010598965 and the construct assembled from consecutive genes). However, the *D. massiliensis* protein assembled from consecutive genes appears far more closely related to the house spider sequence Scaffold901_14 than to its putative *D. massiliensis* homolog.

### Variable venom, sex, and stage-specific expression of latrotoxins

**Fig. 3:**
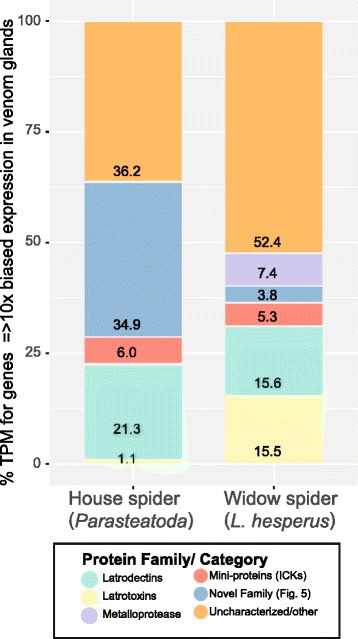
Distinct expression profiles in house and black widow spider venom glands. Shown is the percent of overall expression (as TPM) constituted by five families or functional categories of proteins, including known toxin types, in the venom gland biased transcriptomes (≥10-fold greater in venom than in silk and ovary or cephalothorax tissues and venom expression ≥ 10 TPM) of house (*left* – calculated from 379 venom biased sequences) and black widow (*right* – 417 venom biased sequences) spiders. Mini-protein = inhibitory cystine knot toxins, novel = members of the novel family described in Fig. 5 and Additional file 6, uncharacterized/other = all other proteins in the set of venom gland biased sequences (Additional file 3). Please see Additional file 9 for a high resolution version of this figure

Venom gland expression levels varied among house spider latrotoxin genes, with the highest levels at approximately 1592 Transcripts Per Million (TPM) and 24 of 47 complete genes with TPM values less than 10 (Additional file 1). Transcripts with expression > 10-fold in venom glands versus silk gland and ovary tissues and having venom gland expression ≥ 10 TPM were categorized as venom biased (see Methods). Latrotoxins were not among the 10 most highly venom biased transcripts, which had TPM values ranging from 14168 to 144578 (Additional file 3). We determined that 23 of the 47 complete house spider latrotoxins were venom gland biased. Within the set of 379 house spider transcripts that are venom biased in expression, latrotoxins represent approximately 6% of the unique transcripts and 1.1% of the overall expression (measured in TPM) (Additional file 3). In contrast, among the 417 venom gland biased transcripts identified in *L. hesperus* (black widow) using the same methods, latrotoxins represented approximately 9% of unique transcripts and 15.5% of overall expression (Fig. 3) [22].

The house spider latrotoxins lacking predicted CTDs have little to no expression in female venom glands, but most appear to have greater expression in males (aug3.g21388: venom TPM = 0.21, whole male TPM = 158.72, aug3.g17298: venom TPM = 0.08, whole male TPM = 108.17, aug3.g17299: venom TPM = 0, whole male TPM = 5.24, aug3.g22696: venom TPM = 0.01, whole male TPM = 8.84, Additional file 1). All of the male-expressed latrotoxins form a clade in the phylogeny, though one sequence (aug3.g17298) was not included in the phylogeny because it is interspersed with N’s (Fig. 2). The latrotoxin gene Scaffold901_14, which also lacks a predicted CTD, was expressed at low levels in silk (TPM = 3.84), venom (0.02), and ovary (1.72), with higher expression in whole female (34.77) and whole male (17.75).

### CHH/ITP/latrodectin phylogeny shows shift to venom function involved CHH domain truncation

**Fig. 4:**
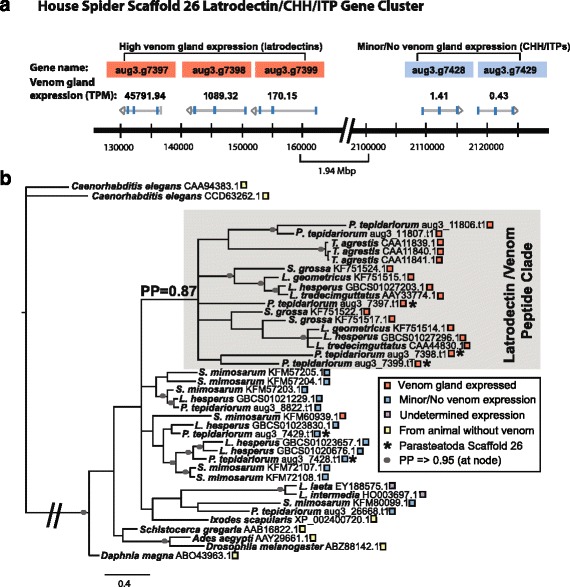
Genomic duplication and inversion of CHH/ITP/latrodectin homologs associated with a shift to venom gland expression. **a** Locations of house spider latrodectin and crustacean hyperglycemic hormone (CHH) and ion transport peptide (ITP) homologs on genomic scaffold 26. Genes with high expression in venom glands are in *orange*, and homologs with low venom gland expression in *blue*; expression indicated by venom gland TPM (**b**) Bayesian 50% consensus tree of diverse CHH/ITP/latrodectin peptides rooted with *C. elegans* ITP homologs, with sequences labeled by species and NCBI accession number. Nodes with posterior probability = > 0.95 indicated by *gray dots*; sequences with high venom gland expressed sequences (venom TPM > 100) labeled with *orange squares*, those with no or negligible venom gland expression (venom TPM < 2) with *blue squares*; those from non-venomous animal with *yellow squares*. Two venom gland ESTs from *Loxosceles* (recluse) spiders that lack abundance/expression estimates are labeled with *purple squares*. The five house spider latrodectins/CHH/ITP peptides encoded on genomic scaffold 26 are indicated with *asterisks. Hatched lines* indicate shortened branch for figure quality. Please see Additional file 9 for a high resolution version of this figure

In contrast to the 47–53 latrotoxins encoded by the house spider genome, we identified only nine putative CHH/ITP/latrodectin genes on four scaffolds. Five of these genes are in tandem on the same genomic scaffold (scaffold 26, Fig. 4a), two are adjacent on scaffold 1379, and the remaining two are on different scaffolds (Additional file 4). Of the nine house spider CHH/ITP/latrodectin genes, all but one (aug3.g11806) of their translations contain the six conserved cysteine residues found in characterized members of this protein superfamily (Additional file 5). Consistent with results from McCowan and Garb [29], all of the house spider CHH/ITP/latrodectin genes have a predicted phase 2 intron interrupting the codon following the fourth cysteine residue, further supporting the derivation of venom latrodectins from the more phylogenetically widespread CHH/ITP neuropeptide hormones.

Five of the nine house spider latrodectin/CHH/ITP homologs appear to have venom-biased expression, two of which have the second and fourth greatest expression in venom glands among all genome predicted genes (TPM = 85741 and 45791, respectively; Additional files 3 and 4), and latrodectins comprise 21.3% of total TPM in the house spider venom gland biased set (Fig. 3). High venom gland expression of latrodectins is also seen in widow spiders ([21, 22]; Fig. 3).

In the Bayesian consensus tree of translated latrodectins and CHH/ITP homologs (Fig. 4b), the five venom-biased house spider sequences form a moderately supported clade (PP = 0.87) with homologs present in other spider venoms or highly expressed in spider venom glands. Included in this clade are venom gland expressed latrodectins from *Latrodectus* and *Steatoda*, as well as venom toxins from the more distantly related hobo spider (*Tegenaria agrestis*). Proteins predicted from venom gland expressed sequence tags (ESTs) from *Loxosceles*, another distantly related spider genus, fall outside of this clade, but their levels of expression relative to other tissues could not be determined. The remaining four house spider proteins with homology to CHH/ITP/latrodectins that have very low venom gland expression (TPM 0–1.41) are also outside of the venom expressed clade. The finding of a venom expressed clade nested among non-venom sequences – including two downstream paralogs on house spider scaffold 26 with low venom gland expression – is consistent with a tandem duplicate of a CHH/ITP gene giving rise to venom expressed latrodectin genes (Fig. 4).

Predicted physiochemical properties of CHH/ITP/latrodectin proteins also support the presence of distinct venom and non-venom/hormone sequence groups. Isoelectric points are below 6.0 for all sequences in the venom clade, while the four house spider sequences outside of the venom clade have predicted isoelectric points greater than 6.0 (Additional file 4). Moreover, the InterProScan predicted CHH domains of house spider non-venom hormone sequences in the phylogeny are 69–74 amino acids long, whereas the CHH domains of house spider venom expressed latrodectins are 51–61 amino acids long. The house spider latrodectin homolog aug3.g11806 has no predicted CHH domain and is among the highest expressed of any genome predicted gene in venom glands (Additional files 3 and 5). However, analysis of the *S. mimosarum* (velvet spider) venom transcriptome yielded one transcript (CUFF.44682.1_Ste) with high venom expression (TPM = 516), and its protein translation (KFM60939.1) has a longer CHH domain length of 74 amino acids.

### Other house spider genes with venom gland biased expression

**Fig. 5:**
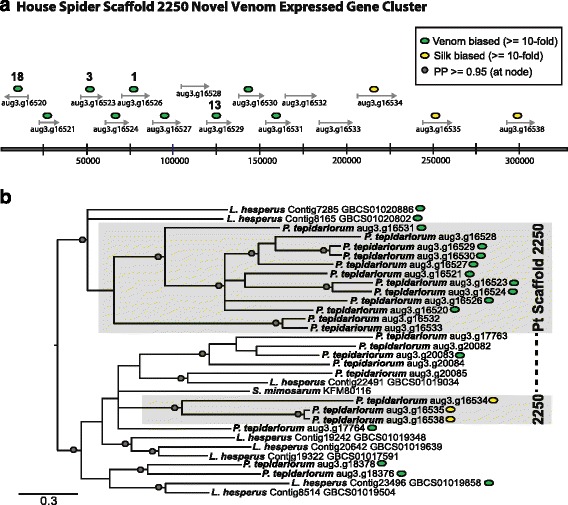
House spider genes with highest venom gland expression compose an uncharacterized family of putative toxins. **a** Tandemly arrayed members of a novel gene family with 10 fold or greater expression in venom glands (*green ovals*) or in silk glands (*yellow ovals*) located on scaffold 2250. Numbers above genes indicate rank in order of highest venom gland expression among all genome predicted genes (e.g., aug3.g16526 had highest venom gland expression, with TPM = 144578). **b** Midpoint rooted Bayesian 50% consensus tree of novel family. Venom gland and silk biased expression labeled as in legend above. *Gray ovals* at nodes indicate posterior probability of = > 0.95. Non-house spider sequences labeled with GenBank accession numbers. Please see Additional file 9 for a high resolution version of this figure

Top BLAST hits of additional house spider venom biased components include small, cysteine-rich mini-proteins (e.g., cystine knot toxins) that BLAST to lycotoxins and ctenitoxins, as well as metalloproteases, which are also found in the venom of *Latrodectus* species, but in different proportions (Fig. 3; Additional file 3, [21, 22]). BLASTclust analysis of translations of the 379 venom biased house spider genes resulted in 30 groups containing three or more sequences. One of the largest BLASTclust group contained 12 sequences, including the most highly venom gland biased transcript (aug3.g16526.t1, venom TPM = 144578). Together, this putative gene family represents 35% of the expression in the venom biased set (Fig. 3). By performing a BLASTp search of the *P. tepidariorum* Augustus predicted protein set using these venom biased sequences as queries and an *e*-value cutoff of 1.00 × 10^−5^, a total of 24 putative family members were identified on four genomic scaffolds, encoding both venom and non-venom biased transcripts. Fifteen of these sequences are tandemly arranged on genomic scaffold 2250, two are on scaffold 2651, three are on scaffold 2831, and four are on scaffold 3606 (Fig. 5a; Additional file 6). While diverse at the amino acid level (ranging from 72 to 171 amino acids in length), nearly all of their encoded proteins contain at least six cysteine residues and a predicted signal peptide motif (Additional files 6 and 7). The only significant BLASTp hit for any of these sequences is to a sequence from the velvet spider *S. mimosarum* (KFM80116).

Bayesian analysis of these sequences was performed using a nucleotide alignment of expressed transcripts with the PAL2NAL software [35], informed with a protein alignment from CLUSTAL Omega (Fig. 5b). Similar to what is observed in the latrotoxin and latrodectin families, the phylogeny of these sequences suggests that substantial lineage-specific expansion has occurred within this family, resulting in many diverse coding sequences with distinct expression profiles. Fourteen of these sequences are venom gland biased and three of the genes (aug3.g16534, aug3.g16535, and aug3.g16538) are silk gland biased at > 10-fold levels relative to venom and ovaries. These three silk biased transcripts are present on the same genomic scaffold as 12 other family members, including several venom biased and highly venom expressed transcripts, but form a separate clade in the phylogeny (Fig. 5; Additional file 6). Three of the five putative *L. hesperus* homologs (Contig23496, Contig7285, and Contig8165) are venom gland biased and two of these transcripts form a clade with a group of venom biased house spider sequences [22]. In contrast to house spider venom in which 35% of the venom gland biased group is represented by this family, homologs represent only a small proportion (approximately 3.8%) of the venom biased transcripts in widow spider venom (Fig. 3).

## Discussion

Because the common house spider (*Parasteatoda tepidariorum)* and black widows are members of the same family, the newly sequenced house spider genome is an important resource with which to identify genomic differences that may contribute to the exceptionally potent venom of black widows. We focused our evolutionary investigation on two families (latrotoxins and latrodectins) that are especially abundant in black widow venom and are also poorly understood due to their narrow phylogenetic distribution. As a result, we identified a multitude of novel genes coding for putative venom toxins, significantly expanded the known members of the latrotoxin and latrodectin gene families, and discovered a novel venom gene family that dominates house spider venom gland expression.

Latrotoxins are predominant contributors to black widow venom toxicity, and α-latrotoxin in particular is associated with extreme vertebrate toxicity [14]. Prior to 2013, four homologs of the latrotoxin family were known, and only from black widow species [14]. We found that the house spider genome includes at least 47 latrotoxin genes and recent black widow transcriptomes have suggested a minimum of 20 latrotoxins (and likely more) in their genome, but we could not identify definitive latrotoxin homologs in the only other high quality spider genome (*Stegodyphus mimosarum*). This high number of latrotoxins in theridiids but not the far more distantly related *S. mimosarum* in the spider family Eresidae is consistent with a relatively recent origin of the latrotoxin gene family in spiders. Since the most recent common ancestor of *Latrodectus* and *Parasteatoda* (~90 million years ago;[34]), the latrotoxin gene family appears to have undergone substantial expansion and diversification, exhibited by the large house-spider specific sequence clades. Notably, no ortholog for the vertebrate specific α-latrotoxin found in *Latrodectus* and *Steatoda* species was discovered in the house spider genome, also suggesting a more recent origin for α-latrotoxin and providing a partial explanation for the lower toxicity of house spider venom relative to widow venom [10].

The greater toxicity of *Latrodectus* venom to vertebrates in comparison to house spider (*P. tepidariorum*) venom is likely due to ecological differences between these species. *Latrodectus* females have larger body lengths (6–13 mm) than *P. tepidariorum* females (5–8 mm) [36–39]. While *P. tepidariorum* and *Latrodectus* are primarily generalist predators of arthropods (multiple insect orders, centipedes, isopods, other arachnids), webs built by *Latrodectus* females are far larger than *P. tepidariorum* webs, which enables *Latrodectus* to occasionally capture and feed upon small vertebrates such as geckos, small lizards, snakes and mice [40–42]. This would suggest that the greater vertebrate toxicity of *Latrodectus* venom is an adaptation to immobilize larger and phylogenetically divergent prey like small vertebrates, whereas toxicity to humans is an incidental by-product. It is also possible that *Latrodectus* venom provides defense from vertebrate predators given the aposematic coloration of *Latrodectus* females with varying levels of bright red coloration on a black abdomen [43]. By contrast, female *P. tepidariorum* have a brown-grey cryptic coloration.

Differences in the ecology of *Latrodectus* and *P. tepidariorum* may also explain the phylogenetic distribution of α-latrotoxin. Within Theridiidae, α-latrotoxin is only known to occur in *Latrodectus* and its sister genus *Steatoda*. Thus it evolved in the common ancestor of these two genera, or earlier, but after the *Parasteatoda/Latrodectus* split. Since its origin, α-latrotoxin may have evolved towards greater vertebrate toxicity in the genus *Latrodectus*, given the genus’ large females that occasionally consume small vertebrate prey, and because *Latrodectus* has evolved aposematic coloration [44]. Determining the origin of α-latrotoxin will require greater sampling of genomes and venom gland transcriptomes from additional theridiid genera. Moreover, functional assays of α-latrotoxin from genera outside of *Latrodectus* would be needed to determine when it acquired vertebrate toxicity.

Our phylogeny, along with the significant similarity in nucleotide sequences between putative *P. tepidariorum* and *Diplorickettsia massiliensis* latrotoxin homologs, supports the hypothesis that the latrotoxin family has experienced a lateral gene transfer event between an ancestor of the house and widow spiders and a bacterial endosymbiont [23]. Interestingly, the sequence similarity that we observed between spider and bacterial sequences occurs in the N-termini and ankyrin repeat regions of the putative homologs from spiders and *Diplorickettsia*, whereas the similarity observed by Zhang et al. [23] and by Bordenstein and Bordenstein [45] occurs in the protein C-terminal domain (CTDs) of black widow spider latrotoxins and arthropod endosymbionts *Rickettsiella* and *Wolbachia*, or their bacteriophages. It is possible that more than one lateral transfer event between spiders and bacteria contributed to the generation of black widow latrotoxin genes. However, the current latrotoxin phylogeny is mid-point rooted (due to a lack of an appropriate outgroup) and positions the *Diplorickettsia* sequences close to the root, which cannot clearly resolve whether the transfer was from bacteria to spider or vice-versa, or when the transfer happened. If this was an ancestral transfer occurring between bacteria and the most recent common ancestor (or earlier) of *Parasteatoda* and *Latrodectus* the transfer would have occurred at least 90 million years ago [34]. However, given the current phylogeny it is also possible that the transfer occurred more recently, after *Parasteatoda* and *Latrodectus* split. Determining the timing and direction of the lateral transfer would require additional genomic data from more theridiid genera*,* which would enable us to root the tree with species tree reconciliation, that might more closely pin-point the time-frame and direction of the transfer.

Garb and Hayashi [44] previously suggested latrotoxins could be endogenous paralogs of transient receptor potential cation channel subfamily A member 1 (TRPA1), a calcium permeable transmembrane channel primarily expressed in sensory neurons that is composed of many ankyrin repeats. However, TRPA1 does not contain the unique N-terminal domain or CTD of latrotoxins and the *P. tepidariorum* TRPA1 ortholog contains many more introns in its gene structure in comparison to the largely intron-less latrotoxin genes. Thus, the evolutionary link between TRPA1 and latrotoxins appears less likely than a potential bacterial origin of latrotoxins, or their origin from a different eukaryotic gene.

Various structural features predicted in house spider latrotoxins, including variable numbers of ankyrin repeats, transmembrane, signal peptide, and t-SNARE domains, suggest that these proteins are functionally diverse. The lower number of ankyrin repeats in house spider venom biased latrotoxins in comparison to that of widow spiders may affect toxicity, as ankyrin repeats mediate protein-protein interactions [14, 46]. Fewer ankyrin repeats in latrotoxins may prevent tetramerization, decrease the efficiency of latrotoxin-membrane receptor binding, or alter interactions between latrotoxins and other venom components, such as latrodectins. The protein translations of all latrotoxin homologs expressed at higher levels outside of our venom gland transcriptome lack latrotoxin CTDs and are most highly expressed in whole male tissue. We hypothesize that these male expressed latrotoxins could be venom components, as previous studies of snakes and spiders have shown sex-specific differences in venom composition [47–50]. However, it is also possible these latrotoxins function in other male-specific tissues such as testes, which we could not determine, as the expression data was derived from whole males.

Our results provide novel evidence to support the derivation of latrotoxin-associated latrodectins from the non-venom CHH/ITP neuropeptide hormone superfamily [25, 29, 30]. It appears that a segmental genomic duplication and inversion event in an ancestor of *P. tepidariorum* led to the current genomic arrangement of tandem latrodectin and CHH/ITP genes, and this coincided with a change in expression of the CHH/ITP genes from body to venom tissue. This pattern of gene duplication and inversion being associated with altered gene expression across the inversion boundary has been documented in prokaryotes and in *Drosophila* [51, 52]. Conserved cysteine codons, which confer structural stability, along with truncation of the CHH domain of CHH/ITP genes may have predisposed them for expression as venom components [53]. Protein domain truncation is associated with increased toxicity in the venom metalloproteinases of snake venom [54], and the CHH domain in some CHH/ITP/latrodectin peptides has convergently become further truncated (relative to black widow venom latrodectins) in the highly insecticidal HAND toxins of *Tegenaria* spiders and centipede venom [30]. In theridiid venom, latrodectins may act in conjunction with latrotoxins to enhance excitotoxicity, or, similar to *Tegenaria* homologs, they may have inherent toxicity [30, 55].

Previous protein gel analyses and bioassays of house spider venom suggested that high molecular weight components (>50 kDa) are primarily responsible for its toxicity in cockroaches [31]. These house spider venom proteins appear to block neuronal signaling rather than causing massive neurotransmitter release at synaptic junctions as caused by latrotoxins. Moreover, the largest venom proteins identified by Young et al. [31] appeared to be <90 kDa, and were smaller than the masses we predict for house spider latrotoxins. It is possible that latrotoxins are selectively expressed under specific environmental conditions or that they are proteolytically cleaved to yield smaller active molecules in venom. However, in comparison to other house spider venom biased genes, latrotoxins are expressed at far lower levels, which is consistent with their apparent absence in the house spider venom protein gel seen in Young et al. [31]. Instead, we found that small molecular weight components, including putative inhibitory cystine knot toxins, latrodectins and other unclassified cysteine-rich venom proteins are far more highly expressed in house spider venom glands, and are likely to be major contributors to venom toxicity. Specifically, over one third of the gene expression from house spider venom gland biased sequences is contributed by members of a novel gene family encoding cysteine rich proteins which are likely to represent venom toxins.

## Conclusions

In summary, our investigation of the house spider genome has substantially expanded the known repertoire of two enigmatic black widow venom toxin gene families (latrotoxins and latrodectins). The latrotoxin gene family, encoding the major toxic elements of black widow spider venom, has at least 47 paralogs in the house spider genome, but remains phylogenetically restricted to a single spider family (Theridiidae) and potentially to endosymbiotic bacteria. Though latrotoxins may be related to bacterial proteins, most venom toxins are generally thought to originate from non-venom genes found within the same genome [53], as we demonstrate with latrodectins being derived from a neuropeptide hormone family. Both expression and past proteomic data suggest major differences in the composition of black widow and house spider venom in terms of the abundance and diversity of latrotoxins and other predicted toxic proteins, implying that these differences have resulted from substantial changes in toxin genomic sequences and/or expression. Accordingly, this study highlights the dynamic patterns of gene duplication, shifts in gene expression, and changes in protein domain organization that can rapidly modify the arsenal of toxins found among the venoms of closely related species and contribute to the evolution of extreme toxicity.

## Methods

### Sequencing of the *P. tepidariorum* genome and transcriptomes

The 1.4 Gb *P. tepidariorum* genome was sequenced at the Human Genome Sequencing Center at Baylor College of Medicine (BCM) as part of the i5k Initiative (NCBI accession GCA_000365465.1; BioProject Accession PRJNA316108). Automated annotation of this genome was performed by the i5k consortium using the Augustus protein prediction pipeline [56] trained with *P. tepidariorum* 454 sequencing of embryonic stages and Illumina RNA-Seq data (NCBI Accession: SRX644660 and SRX646319) including 625 million 100 bp paired end reads from two separate cDNA libraries: the first from whole embryos in stages 1–14 and the second from whole nymphs, a whole male, and a whole female [57]. Additional *P. tepidariorum* Illumina RNA-Seq reads from ovaries, whole males, and whole females were generated by the i5k consortium at BCM (Accession SRX895868, SRX895866, SRX895867).

For this study, we further generated two RNA-Seq libraries: one from female *P. tepidariorum* venom glands (a combination of 32 individuals to obtain sufficient RNA) and the other library from the combined silk gland tissue of a single female. RNA was extracted and cDNA synthesis was performed using protocols from Garb and Hayashi [58]. Venom and silk libraries were constructed using the TruSeq library kit, fragmenting cDNAs into 300-350-nt length pieces. Each library was sequenced on a single lane of an Illumina HiSeq 2000 using 100 bp paired end reads. Reads were trimmed of Illumina adapters and filtered by quality score with Trim Galore v. 0.3.1. Final read counts were 152296364 for venom glands and 162678769 for silk glands.

### Estimating genome-predicted transcript expression

The RSEM software package was used to quantify gene expression from all available tissues, sexes and developmental stages, using the *P. tepidariorum* Augustus predicted mRNA transcripts as a reference database for read alignment [59]. Manual sequence adjustments were made for 3 of the Augustus predictions prior to running RSEM (Additional file 8). Only matched paired-end reads were used in this analysis. For each RNA-Seq dataset, expression levels were estimated in transcripts per million mapped reads (TPM) [59]). Expression of each transcript was compared across venom, silk, and ovary tissues by calculating fold change expression in one tissue versus another (e.g., dividing the venom TPM by the silk TPM). Genes with expression ≥ 10-fold in one tissue relative to the two others and with TPM ≥ 10 were categorized as tissue biased; genes with TPM of 0 in silk and ovary tissues were considered venom biased if their venom gland TPM was ≥ 10. RSEM was also used to map *L. hesperus* venom gland, cephalothorax and silk gland RNA-Seq reads and *S. mimosarum* venom RNA-Seq reads to their respective transcriptomes in order to identify transcripts with high venom gland expression.

### Characterization of genomic latrotoxins, latrodectins and their homologs

A BLAST database was created from the *P. tepidariorum* genomic scaffolds within which putative latrotoxin sequences were identified using a tBLASTn search with a query of the first 320 amino acids of eight divergent *Latrodectus hesperus* latrotoxin proteins from Haney et al. [22]. Latrotoxin queries were restricted to this region because it precedes the ankyrin-rich repeat region that retrieves spurious BLAST hits. The EMBOSS getORF program [60] was used to translate open reading frames (ORFs) ≥600 bp from scaffolds containing significant hits (*e*-value < 1.00 × 10^−5^) to latrotoxins (full-length latrotoxins exceed 4000 bp). The getORF protein sequences were used in a BLASTp search against the NCBI non-redundant protein database (nr) and those with significant hits to known latrotoxins were retained. BLASTp searches for latrotoxins were also performed on a database created from the Augustus predicted proteins from the *P. tepidariorum* genome using queries of: (1) translations of the first 320 amino acids of the N termini of latrotoxins identified in the *P. tepidariorum* genome, (2) translations of the first 320 amino acids of latrotoxins from *Latrodectus* and *Steatoda* species (a putative sister genus of *Latrodectus*), and (3) the C-terminal domains (CTDs) of *P. tepidariorum* latrotoxins predicted using the methods mentioned above. Augustus predicted gene start and end sites were recorded for the final set of *P. tepidariorum* latrotoxins unless the gene was not predicted by Augustus, in which case the genomic coordinates of the start and end sites for the getORF prediction were used.

We searched for *P. tepidariorum* latrodectin homologs among Augustus predicted proteins from the genome using a BLASTp query of latrodectin and CHH/ITP sequences analyzed in McCowan and Garb [29]. We also searched the recently published velvet spider genome [7] for latrotoxin and latrodectin homologs. In addition, all proteins translated from the set of *P. tepidariorum* venom biased transcripts (the 379 transcripts with venom/silk and venom/ovary fold change being 10 or more and venom gland TPM ≥ 10) were grouped using BLASTClust (ftp://ftp.ncbi.nih.gov/blast/documents/blastclust.html) at a stringency of 35% identity over 35% sequence length to identify the largest venom biased families.

InterProScan 5, which combines multiple protein motif databases, was used to search for conserved protein domains among sequences [61]. CHH domains were predicted using the SuperFamily database [62]. The ProP 1.0 server was used to confirm signal peptide cleavage sites predicted with SignalP in putative toxins (http://www.cbs.dtu.dk/services/ProP/). Isoelectric points and molecular masses of proteins were predicted using the EMBOSS iep and pepstats tools (http://emboss.bioinformatics.nl/cgi-bin/emboss/).

### Sequence alignment and phylogenetic analyses

Protein sequence alignments were performed with the CLUSTAL Omega tool, using the BLOSUM matrix, followed by manual adjustments [63]. The latrotoxin alignment included proteins predicted from the house spider genome and putative homologs found in the NCBI nr database having a significant BLAST hit to the first 320 amino acids of house spider latrotoxins, along with homologs from the *L. hesperus* transcriptome [22]. The latrotoxin alignment used for phylogenetic analysis was largely restricted to the conserved and readily aligned N-terminal domain sequence. *P. tepidariorum* latrodectins/CHH/ITP proteins were aligned with homologs reported in McCowan and Garb [29], as well as additional widow and velvet spider homologs [7]. Bayesian phylogenetic trees were computed using MrBayes 3.2.1 [64], using the CLUSTAL protein alignments. MrBayes was run for 5x10^6^ generations with sampling every 1000 generations. The initial 25% of sample trees produced were discarded as burnin. A mixed amino acid substitution model was used. Midpoint rooting was used for the latrotoxin and novel family Bayesian trees and the latrodectin/CHH/ITP tree was rooted with two *C. elegans* ITPs.

## Additional files

Additional file 1: Table describing house spider latrotoxin sequence features. (XLSX 34 kb)

Additional file 2: Significant BLASTp hits (*e*-value < 1×10^−5^) for protein translations of house spider scaffold 901 transcripts. (XLSX 877 kb)

Additional file 3: List of all house spider venom gland biased transcripts. (XLSX 106 kb)

Additional file 4: Table describing house spider latrodectin and CHH/ITP homolog sequence features. (XLSX 29 kb)

Additional file 5: Alignment of CHH/ITP/latrodectins showing duplication and truncation events associated with shift to venom gland expression. (PDF 141 kb)

Additional file 6: Members of a novel gene family from house spider including highly venom gland expressed sequences. (XLSX 29 kb)

Additional file 7: Alignment of novel gene family members that largely contribute to house spider venom gland expression. (PDF 39 kb)

Additional file 8: Manual adjustments to Augustus predicted house spider latrotoxin sequences. (XLSX 47 kb)

Additional file 9: Figures with High resolution version. (ZIP 1198 kb)

## Abbreviations

CHH: Crustacean hyperglycemic hormone
CTD: Latrotoxin C-terminal domain
EST: Expressed sequence tag
ITP: Ion transport peptide
LMWP: Low molecular weight proteins
NGS: Next generation sequencing
ORF: Open reading frame
RNA-Seq: RNA sequencing
TPM: Transcripts per million
