# Supplementary material for: House spider genome uncovers evolutionary shifts in the diversity and expression of black widow venom proteins associated with extreme toxicity
Source: BMC Genomics. 2017 Feb 16;18:178. doi: 10.1186/s12864-017-3551-7 (PMC5314461; doi:10.1186/s12864-017-3551-7)
Supplement: Supplementary file 5 — Alignment of CHH/ITP/latrodectins showing duplication and truncation events associated with shift to venom gland expression. (PDF 141 kb) [file 12864_2017_3551_MOESM5_ESM.pdf]

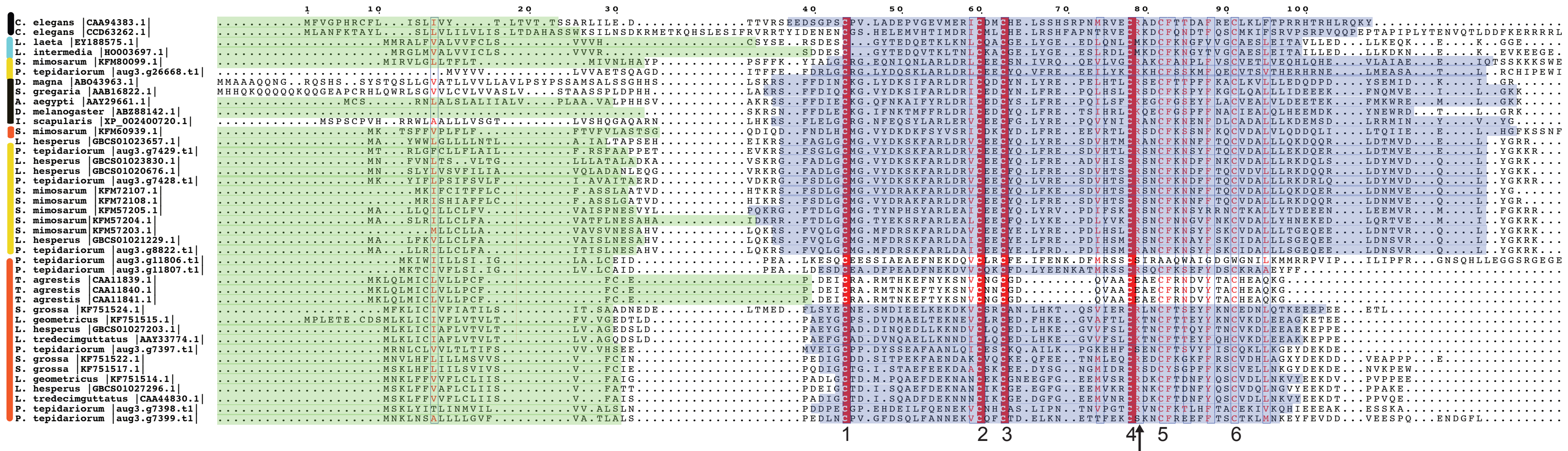

Signal peptide

CHH domain

Venom expressed (TPM>100 in *P. tepidariorum*, *L. hesperus*, *S. mimosarum* venom glands; abundant in *L. geometricus* and *S. grossa* venom gland EST library; or known venom protein)

Undetermined venom expression (from a venom gland EST database with no abundance estimates)

Negligible/ No Venom Expression (TPM <2)

From animal that does not produce venom

Additional File 5: Alignment of CHH/ITP/latrodectin homologs shows that duplication and truncation events are associated with a shift to venom gland expression. All CHH/ITP/latrodectin sequences from *P. tepidariorum* with one exception (aug3.g11806) contain six conserved cysteine residues. The location of a conserved phase 2 intron interrupting the codon following the fourth conserved cysteine residue in each sequence is shown. Levels of venom expression for each sequence is marked by color coded bar on left, with key below. Signal peptides are shaded in green, while CHH domains are shaded purple.
