# Supplementary material for: House spider genome uncovers evolutionary shifts in the diversity and expression of black widow venom proteins associated with extreme toxicity
Source: BMC Genomics. 2017 Feb 16;18:178. doi: 10.1186/s12864-017-3551-7 (PMC5314461; doi:10.1186/s12864-017-3551-7)
Supplement: Supplementary file 7 — Alignment of novel gene family members that largely contribute to house spider venom gland expression. (PDF 39 kb) [file 12864_2017_3551_MOESM7_ESM.pdf]

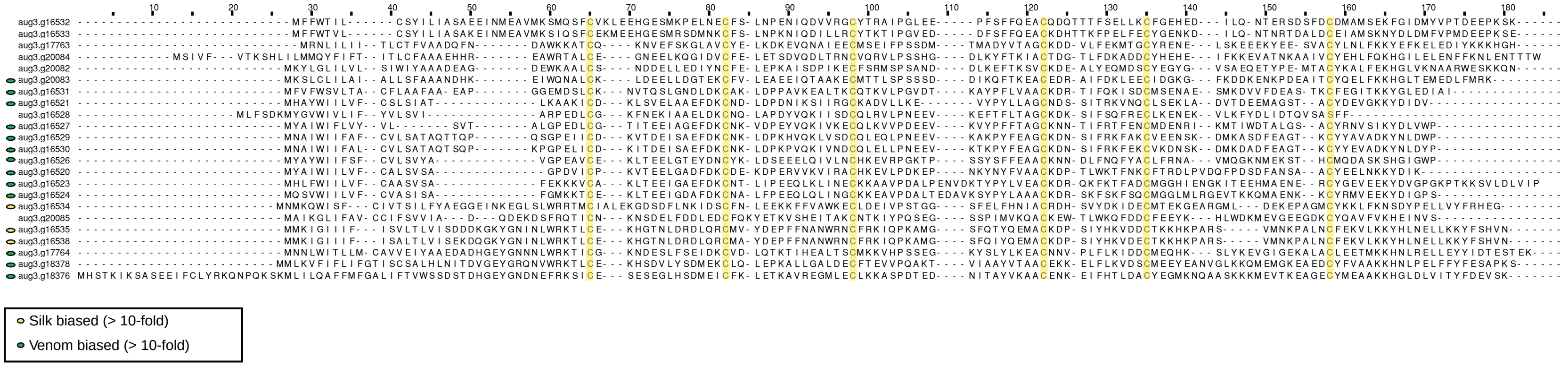

Additional File 7. Aligned translations of members of a putative novel gene family that contribute a signficant proportion of expression in the house spider venom gland. The six conserved cysteine residues in members of this family are highlighted in yellow. The pattern of expression bias is indicated by colored ovals to the left of the sequence label: green=venom gland biased (=> 10 fold), yellow=silk gland biased (=>10 fold).
