# Supplementary material for: House spider genome uncovers evolutionary shifts in the diversity and expression of black widow venom proteins associated with extreme toxicity
Source: BMC Genomics. 2017 Feb 16;18:178. doi: 10.1186/s12864-017-3551-7 (PMC5314461; doi:10.1186/s12864-017-3551-7)
Supplement: Supplementary file 9 — Figures with High resolution version. (ZIP 1198 kb) [file 12864_2017_3551_MOESM9_ESM.zip › 12864_2017_3551_MOESM9_ESM/12864_2017_3551-fig 3.pdf]

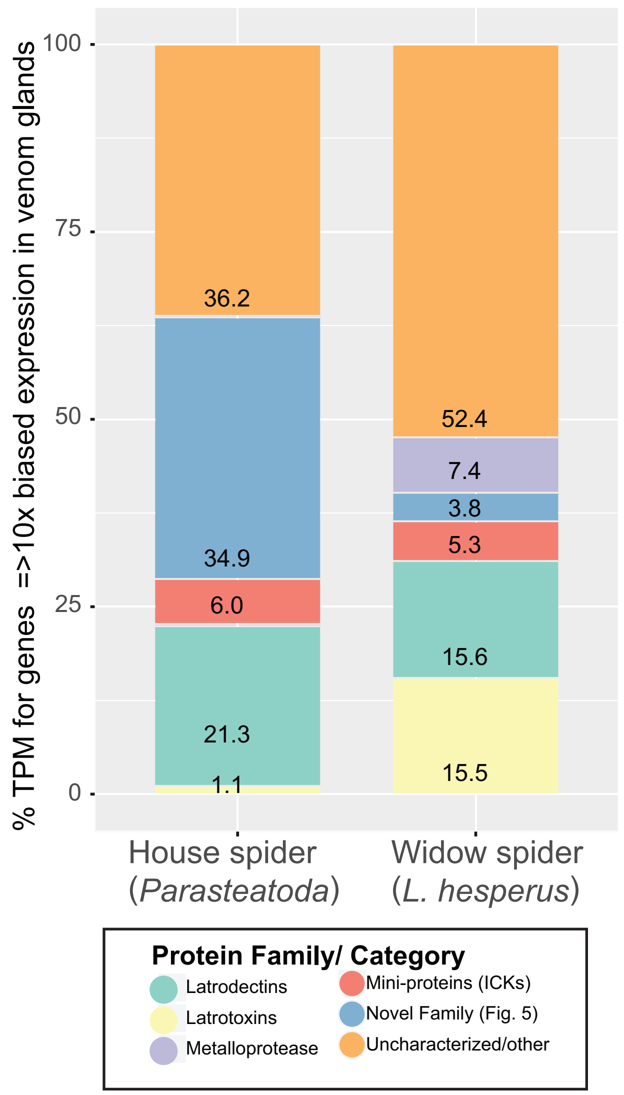

% TPM for genes =>10x biased expression in venom glands
100
75
50
25
0
36.2
34.9
6.0
21.3
1.1
52.4
7.4
3.8
5.3
15.6
15.5
House spider
(*Parasteatoda*)
Widow spider
(*L. hesperus*)
Protein Family/ Category
Latrodectins
Latrotoxins
Metalloprotease
Mini-proteins (ICKs)
Novel Family (Fig. 5)
Uncharacterized/other
