# Supplementary figures and images for: House spider genome uncovers evolutionary shifts in the diversity and expression of black widow venom proteins associated with extreme toxicity
Source: BMC Genomics. 2017 Feb 16;18:178. doi: 10.1186/s12864-017-3551-7 (PMC5314461; doi:10.1186/s12864-017-3551-7)

a. House Spider Scaffold 111 Latrotoxin Gene Cluster

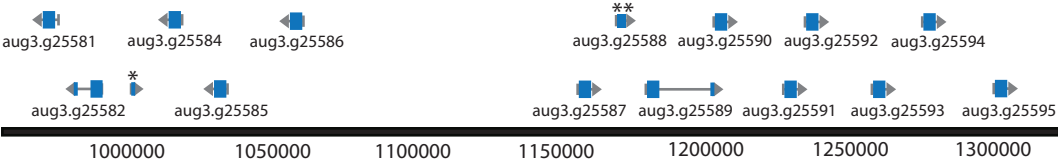

b. House Spider Scaffold 901

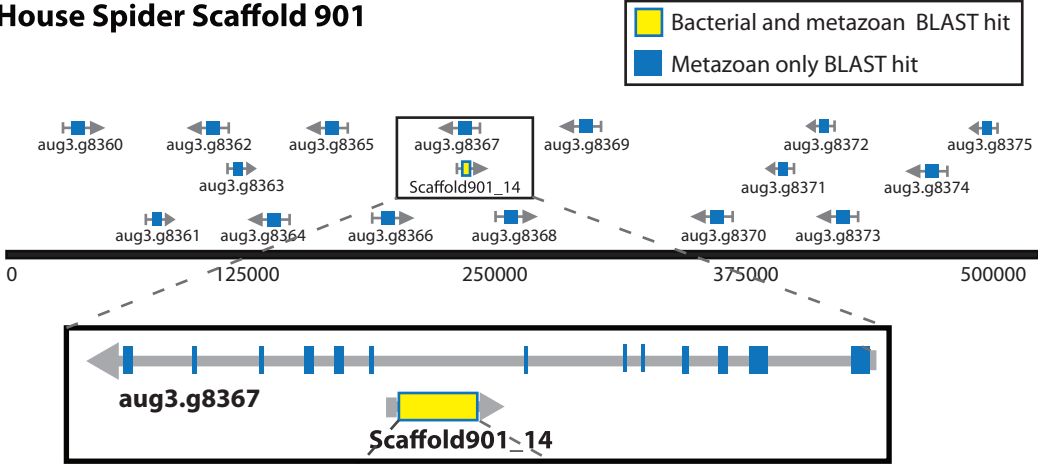

c.

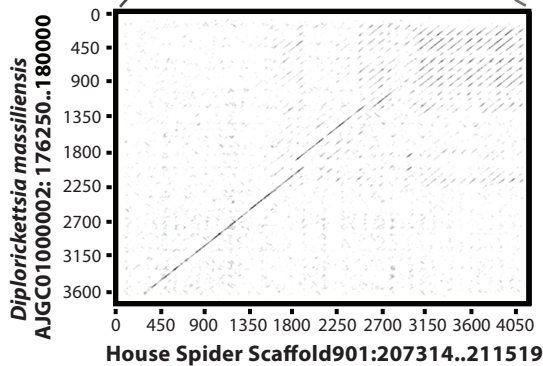

Supplement: Supplementary file 9 — Figures with High resolution version. (ZIP 1198 kb) [file 12864_2017_3551_MOESM9_ESM.zip › 12864_2017_3551_MOESM9_ESM/12864_2017_3551-fig 1.pdf]

# Spider Latrotoxins and *Diplorickettsia* Protein Relationships

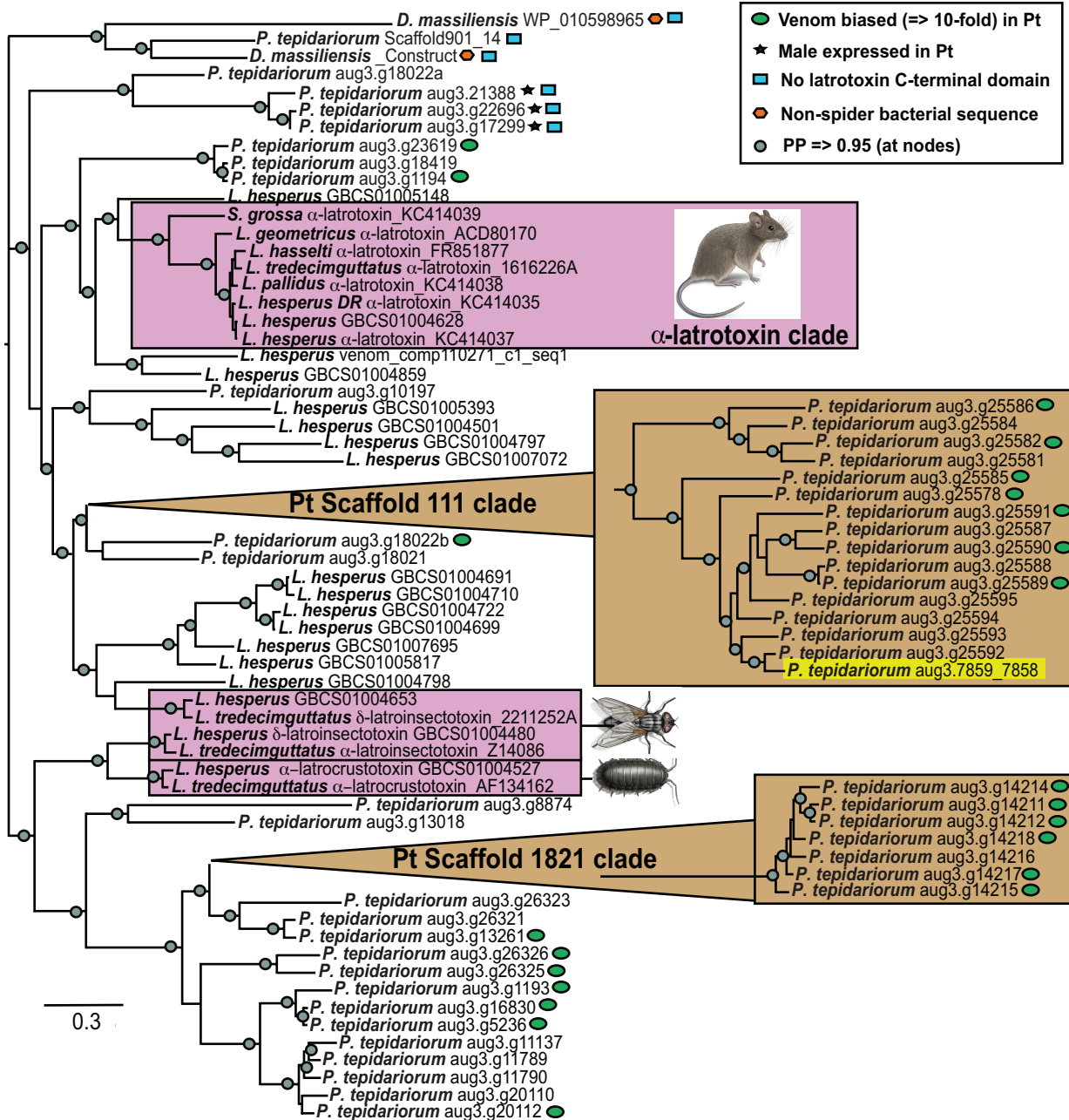

Supplement: Supplementary file 9 — Figures with High resolution version. (ZIP 1198 kb) [file 12864_2017_3551_MOESM9_ESM.zip › 12864_2017_3551_MOESM9_ESM/12864_2017_3551-fig 2.pdf]

a. House Spider Scaffold 26 Latroductin/CHH/ITP Gene Cluster

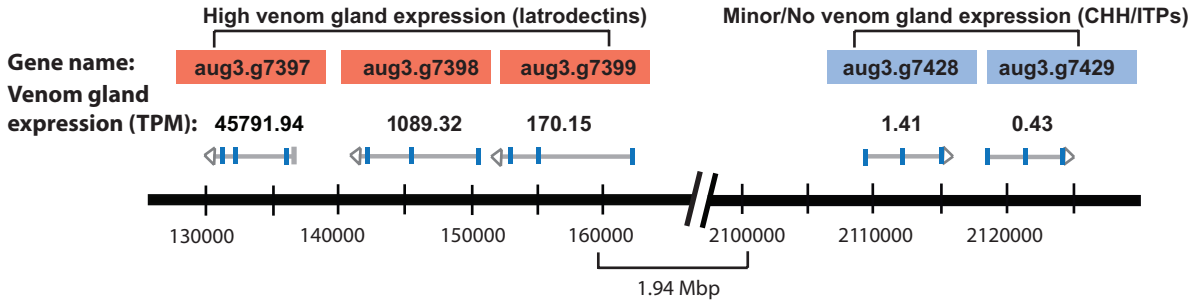

b.

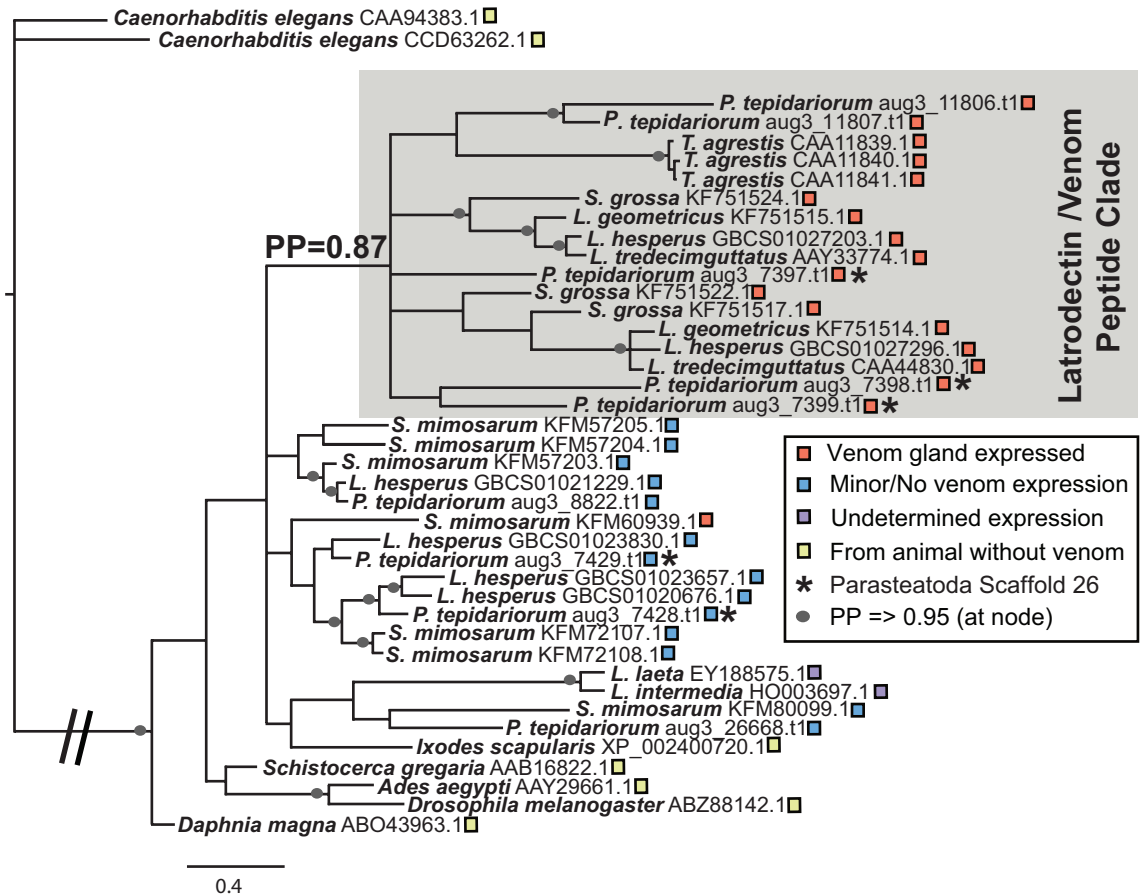

Supplement: Supplementary file 9 — Figures with High resolution version. (ZIP 1198 kb) [file 12864_2017_3551_MOESM9_ESM.zip › 12864_2017_3551_MOESM9_ESM/12864_2017_3551-fig 4.pdf]

## a. House Spider Scaffold 2250 Novel Venom Expressed Gene Cluster

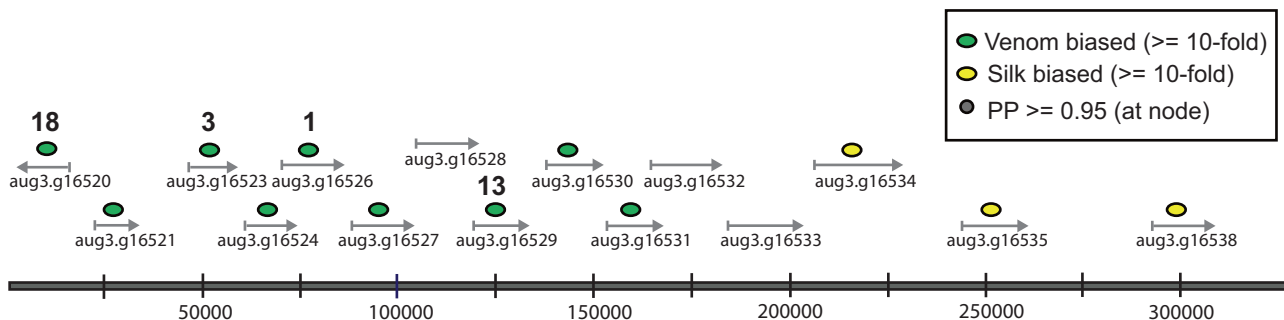

b.

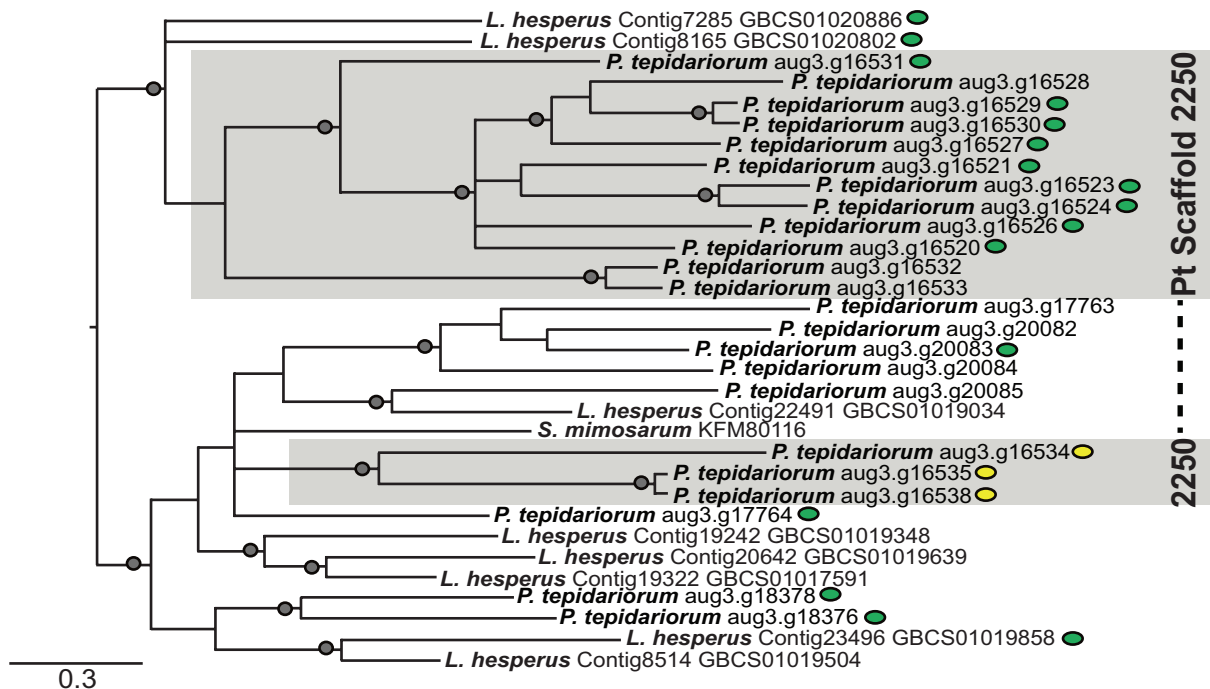

Supplement: Supplementary file 9 — Figures with High resolution version. (ZIP 1198 kb) [file 12864_2017_3551_MOESM9_ESM.zip › 12864_2017_3551_MOESM9_ESM/12864_2017_3551-fig 5.pdf]
